# Dynamically upregulated mast cell CPA3 patterns in chronic obstructive pulmonary disease and idiopathic pulmonary fibrosis

**DOI:** 10.3389/fimmu.2022.924244

**Published:** 2022-08-02

**Authors:** Premkumar Siddhuraj, Jimmie Jönsson, Manar Alyamani, Pavan Prabhala, Mattias Magnusson, Sandra Lindstedt, Jonas S. Erjefält

**Affiliations:** ^1^ Unit of Airway Inflammation, Department of Experimental Medical Sciences, Lund University, Lund, Sweden; ^2^ Medetect AB, Lund, Sweden; ^3^ Division of Molecular Medicine and Gene Therapy, Lund Stem Cell Center, Lund University, Lund, Sweden; ^4^ Department of Thoracic Surgery, Lund University Skane University Hospital, Lund, Sweden; ^5^ Department of Allergology and Respiratory Medicine, Lund University, Skane University Hospital, Lund, Sweden

**Keywords:** mast cells, carboxypeptidase A3, lung, COPD, tryptase

## Abstract

**Background:**

The mast cell-specific metalloprotease CPA3 has been given important roles in lung tissue homeostasis and disease pathogenesis. However, the dynamics and spatial distribution of mast cell CPA3 expression in lung diseases remain unknown.

**Methods:**

Using a histology-based approach for quantitative spatial decoding of mRNA and protein single cell, this study investigates the dynamics of CPA3 expression across mast cells residing in lungs from control subjects and patients with severe chronic obstructive pulmonary disease (COPD) or idiopathic lung fibrosis (IPF).

**Results:**

Mast cells in COPD lungs had an anatomically widespread increase of CPA3 mRNA (bronchioles *p* < 0.001, pulmonary vessels *p* < 0.01, and alveolar parenchyma *p* < 0.01) compared to controls, while granule-stored CPA3 protein was unaltered. IPF lungs had a significant upregulation of both mast cell density, CPA3 mRNA (*p* < 0.001) and protein (*p* < 0.05), in the fibrotic alveolar tissue. Spatial expression maps revealed altered mast cell mRNA/protein quotients in lung areas subjected to disease-relevant histopathological alterations. Elevated CPA3 mRNA also correlated to lung tissue eosinophils, CD3 T cells, and declined lung function. Single-cell RNA sequencing of bronchial mast cells confirmed CPA3 as a top expressed gene with potential links to both inflammatory and protective markers.

**Conclusion:**

This study shows that lung tissue mast cell populations in COPD and IPF lungs have spatially complex and markedly upregulated CPA3 expression profiles that correlate with immunopathological alterations and lung function. Given the proposed roles of CPA3 in tissue homeostasis, remodeling, and inflammation, these alterations are likely to have clinical consequences.

## Introduction

Human adult lungs are densely populated with mast cells at healthy baseline conditions (1, 2). These tissue-inherent mast cells are strategically located throughout all lung compartments where they are thought to have important roles for tissue homeostasis and orchestrating tissue inflammation (3–5). Mast cells have also been implicated in major airway diseases like asthma, chronic obstructive pulmonary disease (COPD), and idiopathic pulmonary fibrosis (IPF) (2, 6–9). In disease, mast cells may act in a pro-inflammatory manner by releasing histamine, leukotrienes, prostaglandins, and cytokines. Another important mechanism by which mast cells influence their tissue surroundings is through their specific proteases. Indeed, the large granules in mast cells serve as storage sites for pre-synthesized effector molecules, with the major content being proteases (10, 11). Among these, tryptase and chymase have been the most studied (10–13) and are also used for the classical division of mast cells into “connective tissue-like” mast cells (MC_TC_ cells; containing tryptase and chymase) and “mucosal- like” mast cells (MC_T_; lacking chymase) (3–5). Less explored is the mast cell-specific zinc-containing metalloprotease carboxypeptidase A3 (CPA3). Still, this protease is equally abundant as tryptase and chymase and has protease activities regulating both local homeostasis and inflammation (14, 15). Among targets for CPA3-mediated cleavage are neurotensin, kinetensin, endothelin-1, apolipoprotein B, and angiotensin I (16–21). It has also been proposed that CPA3 promotes the formation of the fibrous component of the extracellular matrix during tissue remodeling. It thus seems like CPA3 may have both important protective roles and pathogenic pro-remodeling properties.

As support for involvement in disease, increased CPA3 levels have been reported in cancer and cardiovascular diseases (22), diseases of the gastrointestinal tract (23, 24), and the respiratory system (25–27). Altered expression levels of CPA3 have also been proposed as a diagnostic and prognostic marker in asthma and COPD (25–29). Another recent biomarker example is the proposal to use serum CPA3 to monitor severe COVID-19 (30). Although the link between altered CPA3 expression and many clinical manifestations seems clear, very little is known about what effector functions of CPA3 are operating in respective disease.

To gain further insight into the complex roles of CPA3 in health and disease, it is critical to understand the spatial distribution pattern of the mast cell CPA3 expression, as it occurs inside relevant healthy and diseased tissues. Interestingly, recent data suggest that the content of granule-stored CPA3 protein and CPA3 mRNA expression may already under healthy baseline conditions display distinct patterns across human tissues (31). For example, in a recent study (31), a CPA3 protein^high^, mRNA^low^ profile in the skin was contrasted by a reversed CPA3 protein^low^, mRNA^high^ profile in human lungs. This was most dramatic in the alveolar region of normal lungs where the normal chymase-negative alveolar MC_T_ cells commonly lacked CPA3 protein but had a high constitutive CPA3 mRNA expression (31). Based on this observation, and new understanding of fine-tuned gradual mast cell protease secretion mechanisms (14), it has recently been proposed that mast cells may already at healthy baseline conditions exert important homeostatic functions *via* a basal “pacemaker-like” degranulation through microvesicles (14, 31). To what extent this type of basal CPA3 mRNA expression can be further increased in chronic lung diseases emerges as an important question. Since mast cell phenotypes are strongly influenced by the local microenvironments, another critical issue is how the CPA3 mRNA and protein balance is altered in the key anatomical compartments of diseased lungs.

The present study uses a histological approach that yields spatially resolved quantitative data on mRNA and protein within single cells to study how spatially dictated mast cell CPA3 patterns are altered in patients with severe COPD and IPF. The rationale to study COPD and IPF is that, in contrast to the previous important CPA3 studies in asthma (8, 26, 27), little is known about CPA3 in COPD and IPF. Furthermore, from these diseases, as well as controls, it is possible to obtain large enough tissue samples as part of clinical lung surgery and lung transplantation procedures. After exploring such surgical resections, we reveal here novel intriguing observations on altered mast cell CPA3 expression patterns in patients with COPD and IPF.

## Materials and methods

### Human lung tissue acquisition and sample processing for histology

Human lung tissue: Human control lung tissue was obtained from nine non-atopic patients undergoing lobectomy due to suspected lung cancer but otherwise lacking any history of respiratory disease. Only patients with solid well-delineated tumors were included and central and distal lung tissue samples were collected as far as possible from the tumor site. Although most of the subjects are former smokers, this procedure has commonly been used to collect non-diseased control lung tissue samples for histological research (32, 33). Corresponding lung tissues were collected from diseased lungs that we excised as part of a lung transplantation (*n* = 15; nine patients with diagnosed COPD and six confirmed IPF cases). Hence, both the COPD and IPF patients were in the severe category of their respective disease. All COPD patients were in the GOLD 4 severe category and all IPF patients had confirmed progressing lung fibrosis. Basic demographics of the patients are presented in **Table 1**. All 24 subjects gave their written informed consent, which was approved by the ethical committee in Lund, Sweden (Dnr. LU- 2015/891).

**Table:** Table 1. Patient demographics.

Parameter	Lung Controls	COPD Patients	IPF Patients	Overall *p*-value
Subjects, *n* ^#^	9^#^	10^#^	6^#^	
Gender, male/female^#^	3/6^#^	6/4^#^	5/1^#^	
Age, years	74 (64–78) ^#^	62 (59–64)	60 (53–66) ^†^	0.0334
Height, m	1.6 (1.5–1.8)	1.8 (1.6–1.8)	1.8 (1.6–1.8)	0.1347
Body mass index, kg/m^2^	32.7 (25.7–60.2)	30.1 (25.7–35.4)	32.5 (29.2–37.0)	0.2727
Smoking status, never/ex/current	2/6/1	0/10/0	4/2/0	
FEV_1_, L	1.8 (1.7–2.4)	0.8 (0.6–0.8) ^†^	2.0 (1.5–2.5)	0.0002
FEV_1_/(F)VC, %	82 (66–121)	78 (71–88) ^†^	67 (65–70)	<0.0001
FEV_1_, % of predicted	85 (72–95)	22 (18–29) ^†^	67 (44–76)	0.0003
DLCO	88 (65–110)	36 (28–43) ^†^	29 (24–50) ^§^	<0.0001
Corticosteroids (yes/no/unknown)	0/9/0	9/0/1	4/2/0	

Values are median (range) or n. Statistical analysis was performed using Kruskal–Wallis nonparametric test followed by Dunn’s multiple comparison post-test. Chronic obstructive pulmonary disease (COPD); Idiopathic pulmonary fibrosis (IPF); FEV_1_: forced expiratory volume in 1 s; (F)VC: (forced) vital capacity; diffusing capacity of the lungs for carbon monoxide (DLCO). ^#^The mean value of the study group is 68 years. ^†^p = 0.0334 vs. Lung Controls. ^†^p = 0.0002 vs. Lung Controls. ^†^p < 0.0001 vs. Lung Controls. ^†^p = 0.0003 vs. Lung Controls. ^§^p < 0.0001 vs. Lung Controls.

To avoid technical bias inflicted by the variation in fixation times, which commonly occurs in routine clinical pathology laboratories, the excised tissues were prepared at our own research laboratory and histology platform. All tissues were immediately subjected to standardized fixation in buffered 4% formaldehyde (#02176; Histolab, Askim, Sweden) for 24 h ± 4 h at room temperature before being dehydrated in an automated dehydration machine (SAKURA-Tissue-Tek VIP^®^ 6 AI; Sakura Finetek USA, Inc., Torrance, CA, USA) and embedded into paraffin blocks (SAKURA EC350; Sakura Finetek, Inc.). Large multiple cm^2^-sized 4-µm paraffin sections were generated, dehydrated, and used for the present high-end quantitative MC protease analysis.

### Combined *in situ* hybridization and immunohistochemistry (ISH-IHC) for simultaneous detection of CPA3 mRNA, CPA3, tryptase, and chymase protein across mast cells in human lung sections

Initial visualization of CPA3 mRNA was performed through *in situ* hybridization (ISH) using the RNAscope^®^ Multiplex fluorescence kit V2 assay kit (#323100; Advanced Cell Diagnostics, Hayward, CA, USA) for fluorescent ISH (FISH) and using the RNAscope^®^ 2.5 HD Detection Reagents-RED (#322360, Advanced Cell Diagnostics) for chromogenic ISH (CISH), according to the manufacturer’s instructions. Briefly, 4-µm lung tissue sections were baked at 60°C for 1 h and then deparaffinized through a series of alcohol and xylene baths. Afterwards, the slides were incubated with hydrogen peroxide for 10 min at RT and then with mRNA retrieval solution for 15 min at 99°C before dehydration with 99.5% EtOH for 3 min at RT and then evaporation at 60°C for 5 min. After creating a hydrophobic border around each tissue specimen, they were incubated at 40°C for 30 min together with RNAscope Protease Plus and then at the same temperature for 2 h with the CPA3 mRNA probe (#486731). Additional control probes were run in parallel: negative control (DapB, #310043) and positive control probes (PPIB, #486081; Advanced Cell Diagnostics). For fluorescent read-out, tyramide (TSA) amplification was performed with Cyanine 3 (Cy3, NEL744E001KT, Perkin Elmer, Waltham, MA, USA). The slides with fluorescent read-out were then blocked with an HRP blocker and placed in PBS awaiting immunofluorescence staining.

After the FISH of CPA3 mRNA in the Cy3/555 nm channel, slides were subjected to subsequent immunofluorescence immunohistochemical staining for mast cell protein targets such as tryptase (TPSAB1/AB1tryptase and TPSB2/B2 tryptase combined in Cy7/750 nm fluorescence channel) and CPA3 (Cy5/640 nm channel). For tryptases (TPSAB1 and TPSB2) and CPA3 immunostaining, sections were incubated (for 1 h) with a cocktail of a mouse anti-human AB1 tryptase/TPSAB1 (#MAB1222A; 0.1 g/0.1 ml, dilution 1:350; Millipore, Solna, Sweden), mouse anti Human B2 tryptase/TPSB2 (#MAB37961; 1 μg/1 μl, dilution 1:500, R&D Abingdon, UK), and a rabbit anti-human CPA3 primary antibody (#HPA0526634, 0.4 mg/ml, dilution 1:500, Atlas antibodies, Bromma, Sweden).

After a washing step, these primary antibodies were detected by a secondary antibody cocktail (incubated for 1 h) with an AlexaFluor 647 Goat anti-Rabbit secondary antibody (#A27040; 2 mg/ml, 1:200; Thermo Fisher Scientific, Göteborg, Sweden) and an Alexa Fluor Cy7 Goat anti-Mouse secondary antibody (#A21037; 2 mg/ml, 1:200; Thermo Fisher Scientific, Göteborg, Sweden). All the primary and secondary antibodies were diluted in antibody diluent (#S0809, Dako). After completion of combined ISH and IF-IHC staining, the slides were incubated with Bisbenzimide-33342 (#A0741; Applichem, Darmstadt, Germany) for DAPI nucleus counterstaining (358 nm) and mounted with Vectashield (#H1400; Vector Laboratories, Inc.) and No. 1 coverslips (ECN 631-1574; VWR, Radnor, PA, USA).

The present antibody against CPA3 and the other primary and secondary antibodies have been validated and used in multiple previous studies (31, 34, 35). Detailed tests also confirmed the specificity of the staining results, i.e., that there was no artifactual cross-reactions and unwanted streptavidin binding, and that the low autofluorescence within mast cells had no or negligible impact on the analyses.

### Masson Trichrome staining

The serial lung sections (4 μm thickness) from the different study groups (control, COPD, and IPF) were stained with Masson’s trichome (#HT15, Trichrome Stain Kit, MilliporeSigma, Missouri, USA) for evaluation of gross anatomical changes and degree of fibrosis.

### Slide digitalization and IF image quantification

The Olympus VS-200 (Shinjuku, Tokyo, Japan) fluorescence virtual microscopy slide scanning platform was used for digitalization of fluorescence-stained tissue sections. The fluorophores corresponding to DAPI (nuclei), Cy3 (CPA3 mRNA), Cy5 (CPA3 protein), and Cy7 (combined AB1 and B2 tryptase) channels were recorded with “locked” exposure time for each channel across all the samples within an experiment. Separate test experiments confirmed minimal bleeding between the fluorescence filter settings. To measure CPA3 protein and mRNA content at the single-cell level, a tryptase-based mast cell mask was created for mast cells using Qupath Software (version 0.3.0). First, the mask for mast cell regions of interest (ROI) was segmented by applying standard intensity threshold for the tryptase channel (Cy7). Secondly, the generated masks for mast cells from the tryptase channel were digitally superimposed to the raw data images in Cy3 and y5 channels to measure CPA3 the mRNA and protein content, respectively. Finally, after ROI size filtration, the intensity values were corrected by subtracting the tissue background fluorescence value.

To compare protease profiles within defined anatomical compartments, the following structures were manually delineated by cursor tracing: large and small airway compartments (i.e., bronchioles), epithelium and subepithelial tissue, large pulmonary vessels, and alveolar parenchymal regions. The ROIs for all compartments were applied to other channels of the same image to extract the single-cell data, as described above. For spatial visualization of mast cell distribution in lung tissue sections, *x* and *y* coordinates were extracted during the quantification and superimposed on the original image together with color-coded CPA3 intensity levels. *x*,*y*-coordinate-based topographic contour maps and statistical differences in mast cell CPA3 profiles across spatially defined tissue regions were performed using the JMP Software, Version 16, SAS Institute Inc, NC, USA.

### Correlations between mast CPA3 parameters and crude immune cell profiles

To get a crude insight into the type of cellular inflammation associated with the present elevated CPA3, comparisons were made to lung tissue content of key immune cells like eosinophils, basophils, T lymphocytes and B lymphocytes. These immune cells were analyzed by pairwise double immunohistochemistry (eosinophils–basophils and T–B lymphocytes) using the following primary cell identification markers: T cells (a mouse anti-human CD3- #M7254; 138 mg/L; dilution 1:50; Clone F7.2.38, DAKO, Glostrup, Denmark), B cells (a mouse anti-human CD20- #M0755; 126 mg/L; dilution 1:200; Clone L26; DAKO, Glostrup, Denmark), basophils (a mouse anti-human proMBP1 #J175 704, 0.5 mg/ml; dilution 1:150; BioLegend, Täby, Sweden), and eosinophils (a mouse anti-human ECP/clone EG2 #mAB593, 1 mg/ml; dilution 1:700; Diagnostic Development, Uppsala, Sweden). As part of the antigen retrieval process, slides were baked at 60°C for 45 min and pre-treated with low pH heat-induced epitope retrieval (HIER) by a pH6 target retrieval solution (#DM829; Dako, Glostrup, Denmark) in a DAKO PT Link HIER machine (PTlink 200; Dako, Glostrup, Denmark). The immunohistochemical staining was performed in an automated immunohistochemistry robot (Dako Cytomation, Glostrup, Denmark) with Dako wash buffer (#DM831; Dako, Glostrup, Denmark). Briefly, slides were blocked with peroxidase blocking reagent (DAKO-DM821) for 10 min before being incubated with the primary antibody for 1 h, washed, and incubated with a polymer-HRP-labeled secondary ab (#DM822, Envision Flex, Dako, Glostrup, Denmark) for 30 min and brown chromogen development with 3,3′-Diaminobenzidine (# DM827 DAB-Dako, Glostrup, Denmark). Next, the slides were treated with denaturing solution for 5 min (#DNS001L, Biocare, CA, USA) to remove any remaining antibodies for the first target. After the antibody denaturation step, slides were treated with the secondary primary ab for 1 h and polymer-based secondary antibody as above [but with visualization using the green chromogen Vina Green (#BRR807BC, BioCare, CA, USA) for 10 min]. Finally, the slides were counterstained with Mayer’s hematoxylin (#01820; Histolab, Askim, Sweden), mounted with pertex (#00840; Histolab), and digitized using a ScanScope Slide Scanning Robot (Aperio Technologies, Vista, CA, USA). The generated 20X high-resolution images were used for computerized quantitative analysis of chromogens (i.e., immunoreactivity). The amount of immunoreactivity for each cell marker was normalized to the total analyzed tissue area and quantified using the Visiomorph software (Visiopharm, Hørsholm, Denmark).

### RNA sequencing and single-cell analysis

Cell preparations from bronchial biopsies were subjected to single-cell RNA sequencing using the 10x based Chromium system (10x Genomics, USA). Single-cell RNA sequencing libraries were sequenced on Nextseq500 or Novaseq 6000. Raw data were further analyzed using the bustools kallisto pipeline (36). We used the following parameters to filter out low-quality cells: gene and unique molecular identifier (UMI) minimum 1,000/maximum 75,000; percentage mitochondria, minimum 0% maximum 20%; average number of reads/cells, 25,000. All analyses, including log normalization, scaling, clustering of cells, and identifying cluster marker genes, were performed using python packages. We performed principal component analysis dimensionality reduction with scanpy highly variable genes as input. Unsupervised clustering was performed using the scanpy.tl.umap function, the heatmap was generated using the scanpy.pl.heatmap function. Mast cells were identified by the expression pattern of mast cell marker genes. Two outlying cells not shown in the representative UMAP for space purposes but were included for all relevant analyses.

### Data visualization and statistics

All results are presented as median with interquartile range and ranks were compared among samples using the non-parametric Mann–Whitney test. To compare more than two groups of samples, ANOVA test, Kruskal–Wallis test and Dunn’s multiple comparisons test was used. Symbols used for indicating statistical significance: ns, not significant, *p* > 0.05, *p ≤ 0.05, **p ≤ 0.01, and ***p ≤ 0.001. Calculations and graphs were performed using Prism version 6.01 (GraphPad, San Diego, CA, USA). JMP pro 16 (JMP, Cary, NC, USA) statistical software was used for scatterplots and spatially linked contour maps.

## Results

### Differential alteration of lung CPA3 patterns in COPD

**Figure 1:**
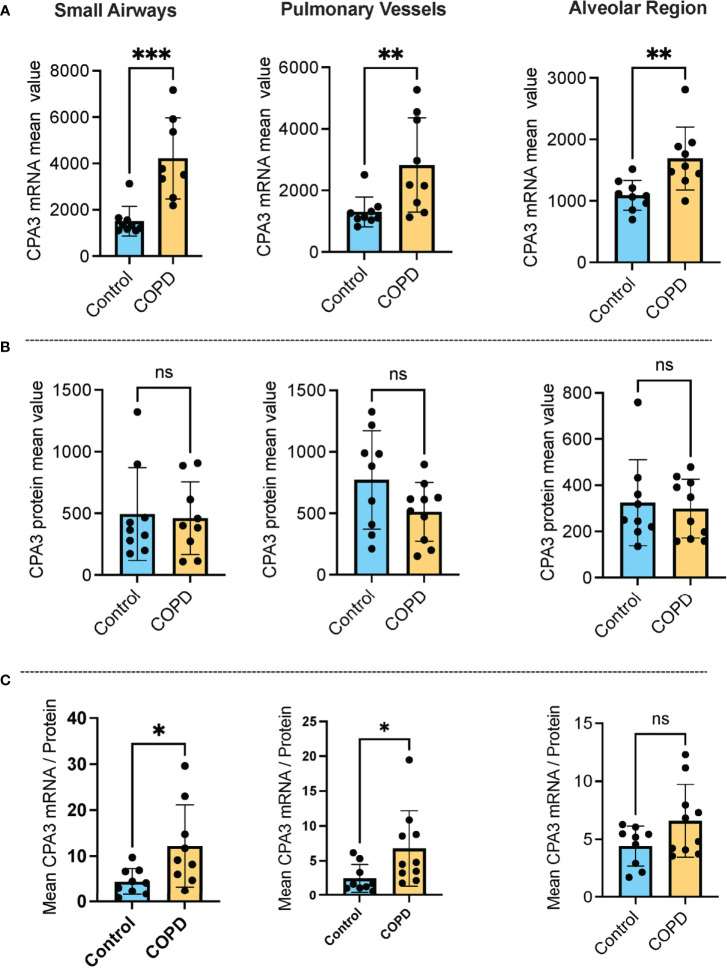
Increased mast cell CPA3 mRNA expression across major lung compartments in COPD lungs. **(A–C)** Mean patient values for individual mast cell expression of CPA3 mRNA **(A)**, CPA3 protein **(B)**, and the mean mRNA/protein quotient **(C)** in control subjects and COPD category GOLD 4 patients. In total, >18,000 individual mast cells were analyzed in the control group and >41,000 mast cells were analyzed in the COPD group. Dots denote mean patient values of >1,500 individual mast cells per control tissue and >2,500 mast cells per COPD patient. Statistical significance levels are marked as ns = non-significant, *p < 0.05, **p < 0.01, and ***p < 0.001.

Our approach for histology-based quantitative analysis of CPA3 expression patterns in individual tissue cells revealed that mast cells in COPD lungs had significant increased CPA3 mRNA expression across the distal lung compartments compared to non-diseased control lungs (**Figure 1A**). The highest numerical and relative increase was seen in small airways and pulmonary vessels where mean patient CPA3 mRNA expression levels increased threefold and twofold, respectively.

Contrasting the mRNA picture, levels of mast cell granule-stored CPA3 protein was not increased in any of the analyzed compartments compared to controls. On the contrary, there was a trend towards reduced CPA3 protein content in pulmonary vessels (**Figure 1B**).

Our simultaneous analysis of CPA3 mRNA and protein allowed for single-cell assessment of the mRNA/protein quotient in all individual mast cells. The mean patient values for this quotient were significantly increased in COPD lungs, particularly in small airways (*p* < 0.05) and pulmonary vessels (*p* < 0.05). A trend towards reduction was also seen in the alveolar parenchyma (**Figure 1C**).

**Figure 2:**
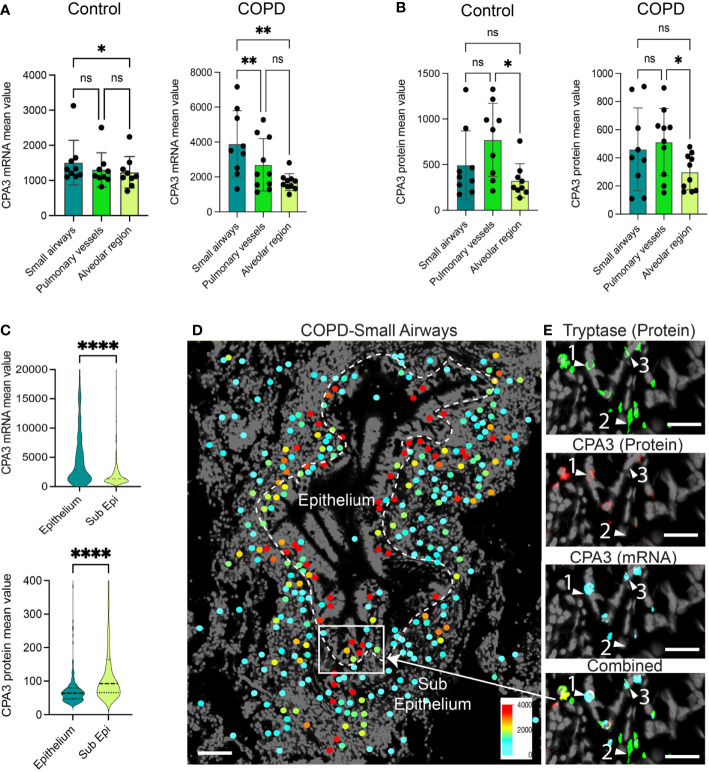
Anatomically differentiated mast cell CPA3 expression responses in COPD lungs. **(A, B)** Mean patient values for individual mast cell expression of CPA3 mRNA **(A)** and CPA3 protein **(B)** across anatomical lung compartments in non-diseased controls and COPD lungs. Statistical significance levels are marked as ns = non-significant, *p < 0.05, **p < 0.01, and ****p < 0.0001. **(C)** Raincloud plots illustrating the difference in CPA3 mRNA and protein expression profiles between small airway intraepithelial and sub-epithelial small airway mast cells. **(D)** Zoomed-in region with a small airway where size-filtered mast cell coordinates have been color-coded for CPA3 level (high = red; low = turquoise). Note the foremost red CPA3^high^ profile in the epithelial region. **(E)** Raw fluorescence images of MC markers exemplifying a CPA3 mRNA+protein double-positive mast cell (label 1), CPA3 mRNA and protein double-negative cell (2), and a CPA3 mRNA-positive and protein-negative cell (3) at an epithelial–subepithelial interface. Scale bars: D = 150 μm, E = 40 μm.

A head-to-head comparison between anatomical sites demonstrated that in COPD, small airway MCs had the highest CPA3 mRNA expression (**Figure 2A**), whereas small airways and pulmonary vessels were equal at the protein level (**Figure 2B**). The discrepancy between CPA3 protein and mRNA content within individual mast cells was also examined within small airway compartments in COPD. Here, intraepithelial mast cells had a striking mRNA^high^, protein^low^ profile compared to the reversed pattern in subepithelial cells (**Figure 2C**). The distinct expression pattern among epithelial and subepithelial MCs is also illustrated in **Figures 2D**, **E**.

### Both CPA3 mRNA and protein increase in IPF lungs

**Figure 3:**
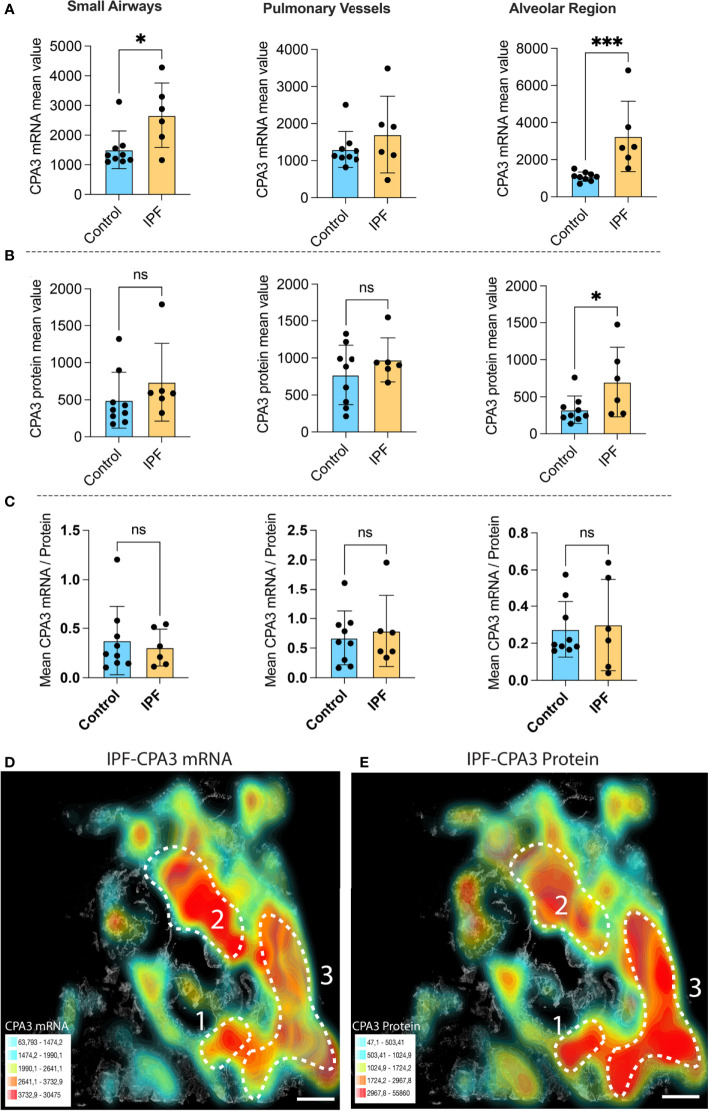
The expanded mast cell population in distal IPF lungs has a robust upregulation of both CPA3 mRNA and protein. **(A–C)** Mean patient values for individual mast cell expression of CPA3 mRNA **(A)**, CPA3 protein **(B)**, and the mean mRNA/protein quotient **(C)** across control subjects and COPD patients. A total of >81,000 individual mast cells were analyzed in the IPF disease group, and each dot denotes mean patient values of >12,000 mast cells per IPF patient. Statistical significance levels are indicated as ns = non-significant, *p < 0.05, and ***p < 0.001. **(D, E)** Low-power micrograph of a representative IPF lung section where a gray-scale background tissue is overlayed with color-coded density maps (high–low is shown as red–turquoise) displaying combined mast cell density and expression levels of CPA3 mRNA **(D)** or CPA3 protein **(E)**. Note that although both the level of protein and mRNA is high, the relative proportion varies across tissue microenvironments, as illustrated by a CPA3 mRNA^high^, protein^moderate^ profile in region 2 and a CPA3 mRNA^moderate^, protein^high^ signature in region 3. Scale bars = 1.8 mm.

**Figure 4:**
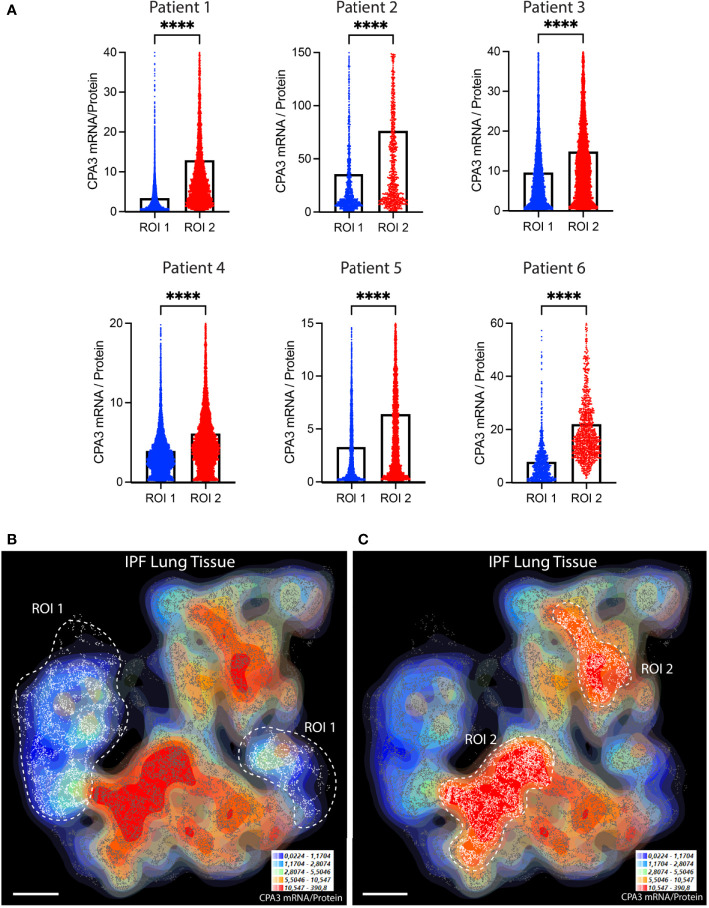
The relative proportion of mast cell CPA3 mRNA and stored protein vary significantly between lung tissue microenvironments. **(A)** Quantitative data and violin plots comparing individual mast cell CPA3 mRNA/protein quotients in pre-determined spatial lung section regions of interest (ROIs). Regions with relatively low (ROI type 1) or high (ROIs 2) quotients were selected from computer-generated topographic contour maps visualizing the heterogeneous spatial distribution of mRNA/protein quotients (exemplified for one IPF section in **(B)** and **(C)**; red denotes spatial regions with a high quotient; coordinates for individual mast cells are shown as white-gray dots). Scale bars: **B**, **C** = 3.5 mm. Black bars in A show mean values and statistical analysis was performed by a Mann–Whitney *U* test and where **** denotes a significance level of *p* < 0.0001.

Compared to control lungs, mast cells in IPF lung parenchyma had a more than threefold significant increase in mast cell CPA3 mRNA expression (**Figure 3A**). Significantly increased levels were also seen in the small airways (**Figure 3A**). Contrasting the situation in COPD, the increased CPA3 mRNA in the IPF lung parenchyma was also accompanied with elevated CPA3 protein levels (**Figure 3B**). A weak trend towards increased mast cell CPA3 protein was observed for small airways (**Figure 3B**). Due to the parallel increased mRNA and protein expression, the mRNA/protein quotient was not increased in IPF compared to controls (**Figure 3C**). Interestingly, although CPA3 mRNA and protein had a parallel increase in IPF, their spatial distribution pattern seemed differentially regulated at a microenvironmental level. One example of this type of dynamic expression heterogeneity is shown in **Figures 3D**, **E**. The spatial heterogeneity and marked variations in relative proportion of mast cell CPA3 mRNA and protein across tissue microenvironments was confirmed statistically by comparing mean mRNA/protein quotients between spatially distinct lung regions within lung sections/patients (**Figure 4**).

### Head-to-head comparisons reveal disease-relevant distinctions in combined mast cell density and spatial mast cell CPA3 expression patterns

The lungs from IPF, COPD, and control groups were subjected to a normalized MC density assessment and in-depth head-to-head MC CPA3 comparisons to gain further insight into the characteristic features of CPA3 expression in the respective disease.

**Figure 5:**
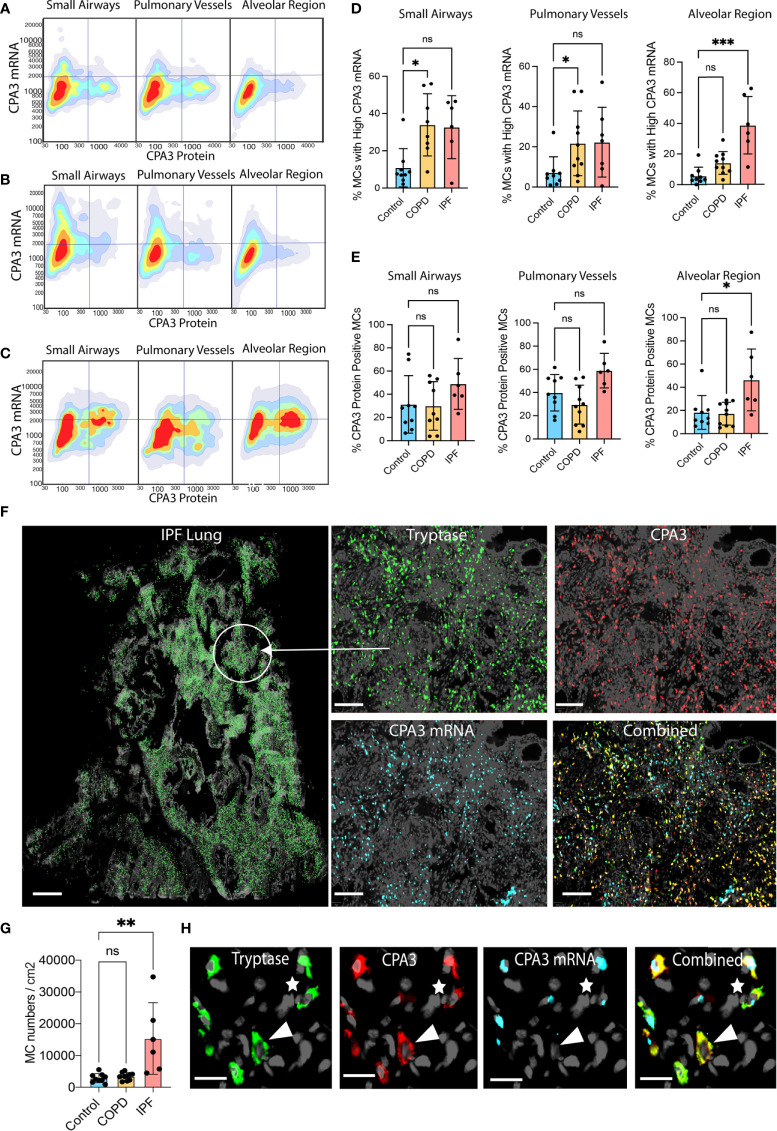
Disease-specific small airway and alveolar CPA3 responses in COPD and IPF. **(A–C)** 2D scattergrams showing CPA3 mRNA and protein relationships across pooled mast cells from control lungs (A; *n* = 18,504 pooled MCs), COPD (B; *n* = 41,313), and IPF cases (C; *n* = 81 806). **(D, E)** Mean patient percentage of lung mast cells with a high CPA3-mRNA profile **(D)** or CPA3 protein **(E)** within anatomical regions and disease categories (as defined by a pre-determined locked threshold values). **(F)** Low-power graph exemplifying the very high density of mast cells in distal IPF lung. To visualize individual mast cells, their *x*,*y* coordinates have been replaced with pseudo-colored green circles (left). The right quartet set of images shows the original fluorescence-based staining in a zoomed-in region. **(G)** Mean patient lung mast cell density levels. Statistical significance levels are marked as ns = non-significant, *p < 0.05, **p < 0.01, ***p < 0.001. **(H)** Zoomed-in ICH and IHC fluorescence images exemplifying a CPA3mRNA^-^, protein^+^ MC (arrowhead) and a CPA3mRNA^+^, protein^+^ MC (* uppermost right corner). Scale bars: H = 25 μm, F-left = 1.8 mm, F-right =200 μm.

Looking at two-dimensional scattergrams visualizing CPA3 mRNA and protein patterns across pooled MCs (>50,000 cells/group), IPF parenchymal MCs stood out by having a unique combined protein and mRNA high signature (**Figures 5A–C**). Interestingly, at this pooled data level, small airway and pulmonary vessel MCs displayed elevated protein compared to controls and COPD (**Figures 5A–C**), although this was not significantly different at a mean patient level (**Figure 3B**). The 2D plots also confirmed the elevated mRNA in COPD, albeit visually less clearly than for the mean patient data in **Figure 1**).

Filtering out only mast cells with a high CPA3 mRNA-high signature demonstrated that in IPF these constituted almost 40% of the total MC pool (contrasting the <5% in non-diseased controls; **Figure 5F**). In the small airways and pulmonary vessels both COPD and IPF had enriched proportions of CPA3 mRNA-high cells compared to controls, although statistical significance was only reached for COPD (**Figures 5D, E**).

Apart from CPA3 expression in individual mast cells, the impact of CPA3 in diseased lungs also depends on the total cell mast cell density. Among the present study groups, COPD lungs had no increase in MC density compared to significant baseline mast cell content in controls (**Figure 5G**). IPF lungs, on the other hand, displayed a striking sevenfold statistical increase (**Figure 5G**). Representative illustrations of this very high MC density and examples of corresponding CPA3 expressions are presented in **Figures 5H**, **I**.

**Figure 6:**
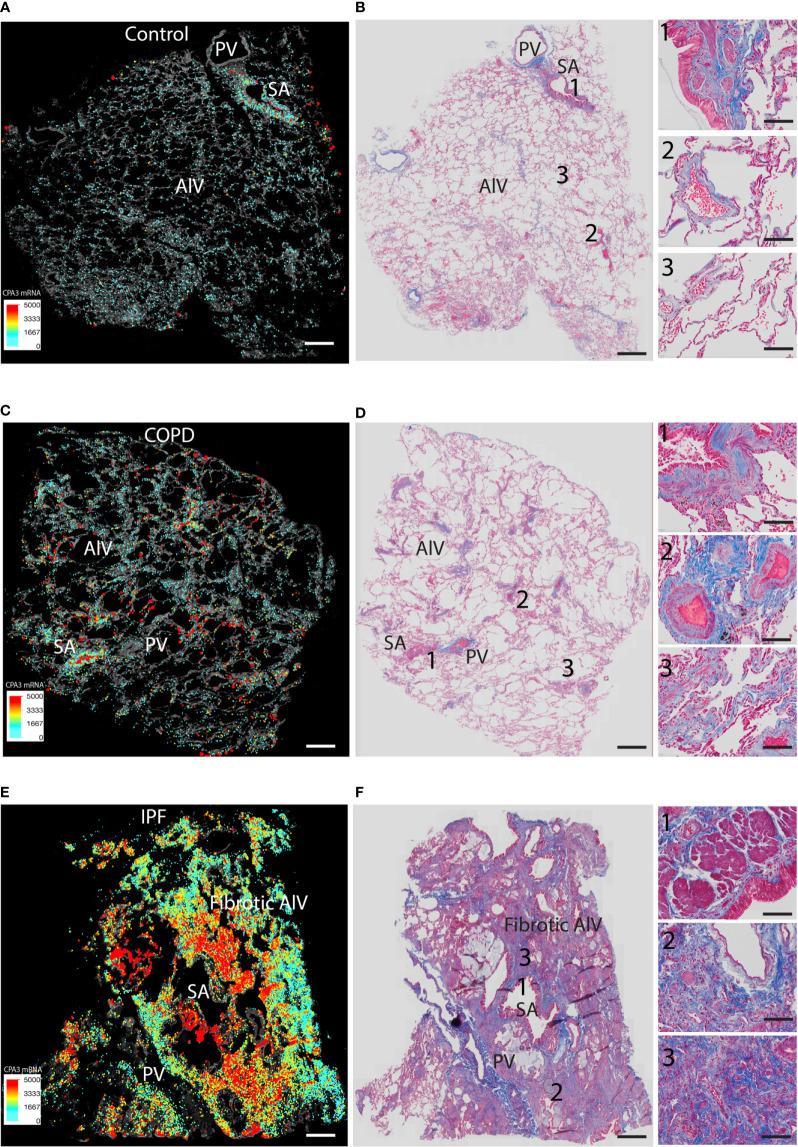
Comparisons of density-weighted and spatial CPA3 variations and lung structure in non-diseased control tissue, COPD lungs, and IPF. **(A, C, E)** Low-power section overviews with gray-scale tissue background in control lung **(A)**, COPD **(C)**, and IPF **(D)** where *x*,*y* coordinates for individual mast cells have been replaced with enlarged dots color and size coded for CPA3mRNA expression levels. **(B, D, E)** Corresponding serial section stained with routine trichrome staining to visualize cells (red) and collagen-rich extracellular matrix (blue) and overall lung structure. SA = small airways; PV = pulmonary vessels; Alv = alveolar parenchyma. The COPD section in **(C**, **D)** show microscopic emphysema whereas the IPF in E and F displays archetype histopathological IPF features like honeycombing, myofibroblast accumulation, and distal parenchymal fibrosis. Scale bars: **A–F** = 1.8 mm, insets in **B, D, F** = 200 μm.

We also performed direct comparisons between color-coded mast cell CPA3 expression maps (providing combined information of MC density and CPA3 expression) and trichrome-stained serial sections (visualizing the structural features and any classical histological features of the lung) (**Figures 6A–F**). This approach confirmed the various degrees of microscopic emphysema and patchy fibrotic foci in COPD lungs and that patchy distal lung regions had elevated CPA3 expression in association with small airways (examples are shown in **Figures 6C, D**). As expected, our IPF sections were characterized by classical IPF histopathological features like parenchymal fibrosis, accumulation of distal lung myofibroblasts and honeycombing (**Figure 6F**) and that these changes were accompanied with a combined striking increase in both MC density and CPA3 expression (**Figure 6**).

### Mast cell CPA3 mRNA correlates to distal lung CD3+ T-lymphocytes and eosinophils

**Figure 7:**
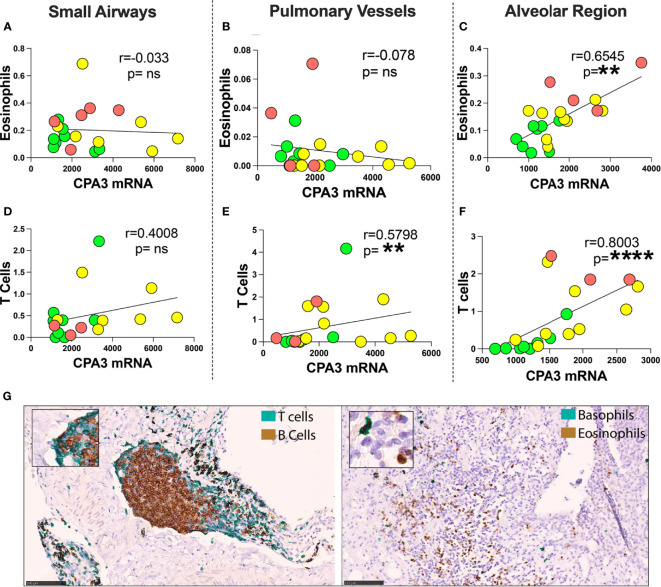
Correlation between mean mast cell CPA3 mRNA and eosinophils and CD3+ T lymphocytes. **(A–F)** Scattergrams with pooled mean patient data (green circles = healthy controls, yellow = COD, and red = IPF). Correlation analysis was performed with the non-parametric Spearman´s pairwise correlation test. *r* = correlation coefficient, the *p*-values and significance levels are shown as **p < 0.01 and ****p < 0.0001. **(G)** Bright-field micrographs exemplifying CD3+ T lymphocyte and CD20-positive B lymphocyte double staining (left) and basophil and eosinophil double staining (right) with brown (DAB chromogen) and green (Vina green chromogen). Scale bars = 100 μm. ns, non-significant.

Comparisons were made between mast cell CPA3 parameters and lung tissue compartment infiltration of eosinophils, basophils, CD3+ T lymphocytes, and CD20+ B lymphocytes. Pooled patient data revealed that for mean CPA3mRNA intensity, there was a statistical positive correlation to alveolar parenchymal eosinophils and CD3+T cells (**Figures 7C, F**). The mean intensity of CPA3 mRNA also correlated to T cells in pulmonary vessel (**Figure 7E**). No statistical correlation was seen in the other compartments (**Figures 7A, B, D**), or between any of the immune cells and CPA3 protein.

### Mast cell CPA3 parameters correlate to several lung spirometry parameters

With the limited number of patients, correlation analysis between the mast cell CPA3 parameters and lung function was made on pooled patient group data (presented in **Table 2**). In summary, the strongest correlations to lung mast cell CPA3 were fond for FEV1, FEV1% predicted and FEV1/VC. Across the lung compartments, the most anatomically widespread correlation was found for pulmonary vessel CPA3 (**Table 2**). Apart from the pooled data, statistically secured correlations between alveolar CPA3 protein/mRNA quotients and FEV1 and FEV1% were also found within the COPD cohort (*r* = −0.84, *p* = 0.004 and *r* = −0.69, *p* = 0.04, respectively).

**Table:** Table 2. Correlations between mast cell CPA3 and lung function parameters.

Mast Cell CPA3	Lung Compartments	FEV1	FEV1%	FEV1/VC	DLCO%
***CPA3 mRNA***	Small Airways	*r* = −0.4168 *p* = 0.0602	***r* = −0.4508** ***p* = 0.0403**	*r* = −0.3449 *p* = 0.1257	*r* = −0.3681 *p* = 0.1459
	Pulmonary Vessels	***r* = −0.5092** ***p* = 0.0155**	***r* = −0.513** ***p* = 0.0146**	***r* = −0.5369** ***p* = 0.0100**	*r* = −0.2097 *p* = 0.4036
	Alveolar Region	*r* = 0.06733 *p* = 0.7659	*r* = −0.1672 *p* = 0.4570	*r* = 0.06337 *p* = 0.7794	*r* = −0.3988 *p* = 0.1012
***CPA3 Protein***	Small Airways	*r* = 0.2748 *p* = 0.2280	*r* = 0.2762 *p* = 0.2256	*r* = 0.2227 *p* = 0.3320	*r* = −0.2038 *p* = 0.4293
	Pulmonary Vessels	***r* = 0.4932** ***p* = 0.0197**	***r* = 0.6736** ***p* = 0.0006**	***r* = 0.6389** ***p* = 0.0014**	*r* = 0.1509 *p* = 0.5500
	Alveolar Region	*r* = 0.2691 *p* = 0.2259	*r* = 0.325 *p* = 0.1401	*r* = 0.2632 *p* = 0.2367	*r* = −0.2749 *p* = 0.2695
***CPA3 Protein/mRNA***	Small Airways	***r* = 0.5766** ***p* = 0.0062**	***r* = 0.666** ***p* = 0.0010**	***r* = 0.4727** ***p* = 0.0305**	*r* = 0.1694 *p* = 0.5123
	Pulmonary Vessels	***r* = 0.5645** ***p* = 0.0062**	***r* = 0.7234** ***p* = 0.0001**	***r* = 0.6786** ***p* = 0.0005**	*r* = 0.1292 *p* = 0.6094
	Alveolar Region	0.2765 *p* = 0.2130	**0.4346** ***p* = 0.0433**	0.2218 *p* = 0.3211	−0.05685 *p* = 0.8227

Spearman r non-parametric correlation analysis was performed using Prism (Version 9.3.1). r = correlation coefficient, p = p value. Statistically confirmed correlations (i.e., p < 0.05) are marked in bold. FEV_1_: forced expiratory volume in 1 s; VC: vital capacity; diffusing capacity of the lungs for carbon monoxide (DLCO).

### Single-cell RNA Seq data

**Figure 8:**
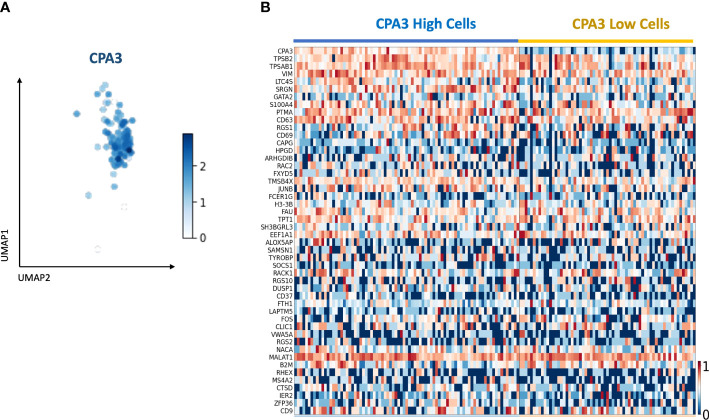
Single-cell RNA sequencing identifies a mast cell population with CPA3 high and low gene expression. **(A)** UMAP of the subset of mast cells determined by the expression of commonly known last cell markers (TPSB2, TPSAB1, MS4A2, and CPA3). CPA3 expression is shown. **(B)** Gene expression heatmap showing cells with “high” and “low” CPA3. Cells were considered “high” when CPA3 expression was above 1.5 and “low” when CPA3 expression was below or equal to 1.5. The genes on the *y*-axis are the top 48 upregulated genes within the mast cell population.

Using single-cell RNA Seq on primary cells isolated from healthy bronchial airways, 148 mast cells were identified based on mast cell-specific gene signatures (**Figure 8A**). Division of the mast cells into CPA3^high^ and CPA3^low^ expressing cells showed a tendency for increased expression of the tryptase genes TPSAB1 and TPSB2 among the CPA3 high MCs (**Figure 8B**). In addition, statistical analysis further suggested that CPA3^high^ profile MCs could be associated with high expression for the leukotriene-producing enzyme LTC4-S, the granule storage-regulating proteoglycan serglycin, and the MC activity regulatory protein annexin-1 (*p* < 0.002).

## Discussion

Carboxypeptidase A3 is one of the most abundant mast cell proteases. It is frequently highlighted in gene expression studies across many diseases. Yet, surprisingly little is known about how the CPA3 mRNA and protein expression patterns are manifested inside diseased patient tissues. Although the present study only involves limited numbers of patients, it offers several new insights into this issue.

Any discussion about disease-modifying functions of mast cells must also consider the mast cell density in the affected organ. In this study, MC density did not increase in the COPD lungs. We have recently shown that in COPD mast cell density in distal lung compartments may not be higher than healthy lungs since an increase in the MC_TC_ population is counteracted by reduced MC_T_ cells (37). The present observation of increased MC numbers in IPF confirms previous observations of elevated mast cell numbers in fibrotic lungs (38–40) and shows that this increase is mainly confined to the fibrotic alveolar parenchyma, a compartment that, in this study, also displayed the most striking alteration in CPA3 expression.

Despite that mast cells have been implicated in most fibrotic diseases, including IPF (2, 9, 41, 42), little is known about their specific role in disease pathogenesis. From the known properties of MC proteases, these roles may be both protective and destructive. An interesting observation in this regard is the report of higher MC density in biopsies from IPF patients being correlated with a slower decline in lung function (40). On the other hand, a more recent study showed that plasma tryptase is elevated in IPF patients and correlates negatively with lung function (FVC), suggesting a pro-pathogenic role (41).

The present findings suggest that the structural pathologies in both COPD and IPF lungs are associated with an elevated local CPA3 mRNA expression. Importantly, the fact that the MC numbers are significantly elevated in fibrotic regions in IPF further increases the local tissue capacity to generate CPA3. Indeed, the present >300% increase in average MC CPA3 mRNA expression and sevenfold increase in MC density create a situation where the total distal lung capacity of CPA3 release is theoretically >20 times ()! higher compared to the already robust CPA mRNA expression at lung healthy baseline.

A similar theoretical exercise may also be done for COPD lungs, although in contrast to IPF, MC numbers are more modest, and the highest relative CPA increase was found in small airways rather than the alveolar parenchyma. Furthermore, COPD patients differed from IPF since they did not have elevated CPA3 protein.

Interestingly, in another COPD study, increased sputum CPA3 was particularly high in female patients with HRCT-defined emphysema (43). Unfortunately, the limited patient numbers in this study prevented us from exploring how the present CPA3 alterations in COPD relate to degree of emphysema.

It is, however, noteworthy that, as for asthma (34, 44), CPA3 levels in COPD and IPF may be particularly elevated in the subcategory of patients that have a type 2 immunity and eosinophil signature (29, 45). Indeed, the present correlation between pooled patient mast cell CPA3 mRNA and eosinophils supports this possibility. Interestingly, CD3+ T cells also correlated with CPA3 mRNA but not protein. Although larger follow-up studies are needed, from other studied linking luminal CPA3 to steroid sensitivity in asthma, it is tempting to speculate that local tissue content of elevated mast cell CPA3 mRNA may be a general marker of both type 2 immunity and steroid responsiveness.

Taken together, our study agrees with previous data on increased sputum CPA3 in COPD (29, 43, 45) and elevated CPA3 in IPF (39). It is likely that the strikingly increased and strategically localized MC CPA3 mRNA expression revealed in this study will impact pathophysiological and immunopathological processes in COPD and IPF lungs. Interestingly, despite the limited patient numbers, we did observe some correlations between elevated CPA3 parameters and worsening of lung function. What is more difficult to speculate on is whether the net effect of CPA3 release is harmful or protective.

Of potential importance for both IPF and COPD is the capacity of CPA3 to regulate smooth muscle constriction, blood vessel tonus, and vascular flow through proteolytic modification of, e.g., endothelin-1, angiotensin I, apolipoprotein B, and neurotensin (46, 47). The capacity of CPA3 to counteract these mediators would likely be beneficial in lung disease, like the protective actions of CPA3 demonstrated in animal models (14, 47, 48). As further support for a potential protective role in relation to the cardiovascular aspects associated with severe COPD and IPF is the observation that decreased serum CPA3 is associated with risk factors of blood vessel disease and cardiovascular damage (49).

Even with the suggested role of CPA3 in protection and homeostasis, the upregulated CPA3 mRNA may also have pro-inflammatory or pathogenic actions. One example of potential relevance for the fibrotic processes in IPF and the small airway remodeling in COPD is the proposed importance of CPA3 for biogenesis of the fibrous component of the extracellular matrix (14, 50). Currently, there are no CPA3 inhibitors in clinical use. Hence, given the multifaceted biological actions of CPA3 protease activity, it thus remains an open question whether pharmaceutical neutralization of CPA3 would have therapeutic benefits or would interfere with homeostatic mechanism for limiting harmful effects of internal pro-inflammatory mediators. More research is needed to identify the tentative good and bad effects of CPA3.

Similar to our recent study on non-diseased lungs (31), the present study shows that also in advanced lung disease, mast cells may have high mRNA expression while displaying a seemingly surprising paucity of granule-stored CPA3 protein. In our study, this phenomenon was most evident in COPD where the mRNA/protein quotient was higher than for controls and IPF patients. The most extreme imbalance was observed in the small airways where intraepithelial and subepithelial mast cells displayed reversed mRNA/protein patterns. Interestingly, a specifically high CPA3 signature in epithelial mast cells have also been reported in type 2 asthma (34).

The discrepancy of CPA3 protein and CPA3 mRNA levels call for cautiousness when predicting biological roles based on only measuring one of these parameters. In this context, the present calculation of mRNA/protein quotients for CPA3 may be a more useful indication of CPA3 turnover in disease than merely study protein or mRNA alone. Especially predicting CPA3 roles based on only CPA3 protein in tissues seems risky, since higher CPA3 levels may merely reflect increased numbers of resting MC_TC_ cells, the subset of mast cells with a high content of co-stored CPA3 and chymase in resting states.

The present observation of striking increases in mRNA/protein quotients in severe disease raises an important question, namely, to what extent is the elevated CPA3 mRNA translated into protein? Furthermore, what is the evidence that CPA3 can be released from mast cells with low content of granule-stored CPA3 but high mRNA expression? Interestingly, recent observations may shed some light into this issue. Firstly, a high mRNA gene expression is coupled to translation and subsequent protein synthesis. Tentatively, a high CPA3 mRNA expression in MCs lacking granule CPA3 may be expected in cells during the refilling phase after degranulation. This could be a possibility in an active disease since we have previously demonstrated that mast cells in COPD, but not in controls, may undergo piecemeal degranulation (37). Classical degranulation, of course, cannot explain the enigmatic high expression of CPA3 and tryptase mRNA already at healthy baseline levels. Here, from new information on the many non-IgE-mediated alternative pathways for degranulation (51, 52), it may be proposed that, as part of a healthy baseline homeostatic regulation, mast cells may constitutively release tryptase and CPA3 directly *via* small secretory vesicles rather than through classical large granules (14, 31). Speculatively, a similar but heightened mode of release may also explain the increased CPA3 mRNA in the present study.

The methodological basis in our study was a histology-based approach using combined ROI masks (in this case, digital mast cell contours) to measure the CPA3 mRNA and protein at a single-cell level directly in routine formalin-fixed paraffin-embedded (FFPE) samples. The high MC numbers in the present large surgical sections, typically 5–12,000 mast cells, provide a robust statistical evaluation for this type of analysis. Naturally, for our approach to be valid, the combined tryptase-chymase ROI masks used for the CPA3 analysis must be mast cell specific. Theoretically, basophils could have affected the measurements since they are known to express tryptase (53, 54). However, both basophil tryptase expression (53, 54) and numbers of basophils (55) are much lower than for mast cells and we have in a recent study showed that they will not be picked up by the staining intensity threshold used for the present computerized tryptase and chymase-based MC segmentation (31).

Another methodological aspect worth noting is that, in contrast to other single-cell staining intensity methods like flow cytometry and high-end single-cell RNA sequencing, the present histology approach analyzes “2-dimensional” cell profiles rather than whole cells. Accordingly, variations in granule and nucleus areas among cell profiles generate some fluctuations of the staining intensity levels. Even so, considering the statistical power of the vast numbers of analyzed mast cells, it seems clear that this phenomenon, which occurs similarly among the sections and study groups, has no relevant impact on the main study conclusions. Another technical perspective is the potential bias of differences in tissue processing. To minimize any such bias, all lung sample tissues in this study were processed at our research laboratory in a consistent fashion, rather than at the routine clinical pathology unit where fixation time, etc. may vary considerably due to logistical issues. Furthermore, the ISH-IHC staining procedures were performed simultaneously for all tissue sections and study groups, resulting in identical processing conditions.

For practical reasons, the patient number was limited in this study. Despite this, given the clear statistical differences between the study groups, it can be argued that the study material is well justified to allow for robust conclusions around the major observations.

Since we used explanted lungs from lung transplantation cases, the COPD and IPF patients were all within the very severe spectrum of their respective disease. Future studies are now needed to explore to what extent the present CPA3 alterations occur earlier during disease progression. That said, in very severe cases, there is patchwork of microenvironments representing different stages of histopathological alterations. Because of their severe stage, both the COPD and IPF patients were subjected to significant steroid treatment, and it cannot be excluded that this have influenced the results.

The major histology-based exploration was in this study complemented with a single-cell RNAseq analysis. The rationale was to get an exploratory first insight into how a more holistic gene expression profile differs between CPA3^high^ and CPA3^low^ mRNA expressing mast cells. In alignment with recent studies (31, 56), and despite the fact that CPA3 is strongly linked to chymase at a protein level (31), CPA3 mRNA expression seemed to be more linked to the tryptase genes TPSAB1 and TPSB2. However, at a single-cell level, the tryptase gene expression seemed more universal across the analyzed MC pool. Although our study material for single-cell sequencing was limited and only involved mast cells from non-diseased human bronchi, it is interesting that high CPA3 expression seems to correlate with heightened gene expression for LTC4S, an enzyme involved in synthesis of proinflammatory leukotrienes. On the other hand, there was a weak trend among the CPA3 high mast cells to have higher expression of Annexin-1, a phospholipid-binding protein with potent anti-inflammatory properties that in mast cells negatively regulates pro-inflammatory actions (57, 58). While these observations serve as good example of the complex and multifaceted pro- and anti-inflammatory roles of mast cells, further studies are needed to explore how these results compare to broader mast cell pools, including MCs from diseased lungs.

In summary, this study has demonstrated that lung tissue mast cell populations in COPD and IPF-affected lungs have spatially complex and markedly upregulated CPA3 expression profiles that correlate with sites of structural pathologies. Given the assumed roles of CPA3 in regulating tissue homeostasis, remodeling, and inflammation, finding out what impact elevated lung CPA3 has on clinical outcomes emerges as an important field for future research.

## Data availability statement

The datasets presented in this study can be found in online repositories. The name of the repository and accession number can be found below: NCBI Gene Expression Omnibus; GSE201772.

## Ethics statement

The studies involving human participants were reviewed and approved by Swedish Research Human Ethics Committee in Lund, Sweden. The patients/participants provided their written informed consent to participate in this study.

## Author contributions

JE and PS conceived and designed the studies, designed the experiments, interpreted the generated data, and wrote the manuscript. JJ contributed to image digitalization. SL contributed to clinical assessment and collection of surgical material. MM and PP planned, performed, and analyzed the single-cell RNAseq experiments. PS and MA performed immunohistochemical analyses and quantitative computerized image analyses. All authors read, provided feedback, and approved the final version of the manuscript.

## Funding

This work was supported by the Swedish Heart and Lung Foundation (20190508) and the Swedish Medical Research Council (VR2017-19). None of the funding sources took part in the planning, execution, or publication of the present study.

## Acknowledgments

We thank Daisy Bornesund at the Unit of Airway Inflammation, Department of Experimental Medicine for skillful technical assistance with tissue processing and serial sectioning.

## Conflict of interest

Jimmie Jönsson is an employee of Medetect AB. Jonas S Erjefält is founder of Medetect AB.

The remaining authors declare that the research was conducted in the absence of any commercial or financial relationships that could be constructed as a potential conflict of interest.

## Publisher’s note

All claims expressed in this article are solely those of the authors and do not necessarily represent those of their affiliated organizations, or those of the publisher, the editors and the reviewers. Any product that may be evaluated in this article, or claim that may be made by its manufacturer, is not guaranteed or endorsed by the publisher.
